# Supplementary material for: Genotype distribution of human papillomavirus among women with cervical cancer stratified by HIV status in Tanzania
Source: PLoS One. 2025 Aug 1;20(8):e0322311. doi: 10.1371/journal.pone.0322311 (PMC12316304; doi:10.1371/journal.pone.0322311)
Supplement: Attachment — Submitted filename: matrix_response_HPV-HIV.docx [file pone.0322311.s002.docx]

**RESPONSE TO REVIEWER’S COMMENTS**

**Title:** **THE DISTRIBUTION OF HUMAN PAPILLOMAVIRUS GENOTYPES AMONG WOMEN WITH CERVICAL CANCER BY HIV INFECTION STATUS IN TANZANIA**

**Corresponding author’s name:** Emmanuel L.Lugina

**Email:** elugina@yahoo.com

| **S/N** | **Comments from the reviewer** | **PI’s response** |
| --- | --- | --- |
| 1 | We've checked your submission, and before we can proceed, we need you to address the following issues:  Thank you for submitting the above manuscript to PLOS ONE. During our internal evaluation of the manuscript, we found significant text overlap between your submission and the following previously published works, some of which you are an author.  <https://www.omicsonline.org/open-access/human-papillomavirus-genotypes-in-invasive-cervical-carcinoma-in-hiv-seropositive-and-seronegative-women-in-zimbabwe-2332-0877-1000343-96252.html?aid=96252>  <https://cytojournal.com/cancer-cervix-epidemiology-and-disease-burden/>  <https://edoc.ub.uni-muenchen.de/30540/1/Mbuya_Wilbert_Laurence.pdf>  We would like to make you aware that copying extracts from previous publications, especially outside the methods section, word-for-word, is unacceptable. In addition, the reproduction of text from published reports has implications for the copyright that may apply to the publications.  Therefore, we cannot consider your manuscript as it stands. Please revise the manuscript to rephrase the duplicated text and fully cite all your sources, where appropriate.  We will carefully review your manuscript upon resubmission, so please ensure that your revision is thorough. | Thank you for this critical comment.  The manuscript has been revised, and the duplicated text has been rephrased.All sources have been fully cited. |
