# Supplementary material for: Genotype distribution of human papillomavirus among women with cervical cancer stratified by HIV status in Tanzania
Source: PLoS One. 2025 Aug 1;20(8):e0322311. doi: 10.1371/journal.pone.0322311 (PMC12316304; doi:10.1371/journal.pone.0322311)
Supplement: Attachment — Submitted filename: HPV Subtypes Matrix responses.docx [file pone.0322311.s003.docx]

**RESPONSE TO REVIEWER’S COMMENTS**

**Title:** GENOTYPE DISTRIBUTION OF HUMAN PAPILLOMAVIRUS AMONG WOMEN WITH CERVICAL CANCER STRATIFIED BY HIV STATUS IN TANZANIA

**The corresponding author’s name**: Emmanuel L.Lugina

**Email: elugina@yahoo.com**

| **S/N** | **Comments from the reviewer** | **PI’s response** | **Page No.** |
| --- | --- | --- | --- |
| **Journal Requirements** | | | |
| 1 | Please ensure that your manuscript meets PLOS ONE's style requirements, including those for file naming. The  PLOS ONE style templates can be found at  https://journals.plos.org/plosone/s/file?id=wjVg/PLOSOne_formatting_sample_main_body.pdf and  https://journals.plos.org/plosone/s/file?id=ba62/PLOSOne_formatting_sample_title_authors_affiliations.pdf | The manuscript has been organized to meet PLOS ONE’s style requirements. | NA |
| 2 | “The authors are grateful for the seed funding program of the US National Institute of Health U54 CA190155 grant.  (C.W.), which supported this study.”  Please state what role the funders took in the study. If the funders had no role, please state: "The funders had no  role in study design, data collection and analysis, decision to publish, or manuscript preparation."  If this statement is incorrect, you must amend it as needed.  Please include this amended Role of Funder statement in your cover letter; we will change the online submission  form on your behalf. | The funders had no  role in study design, data collection and analysis, decision to publish, or preparation of the manuscript  The amended role of the funder statement has been included in the cover letter. | 19 |
| 3 | We note that your Data Availability Statement is currently as follows: [All relevant data are within the manuscript  and its Supporting Information files.]  Please confirm at this time whether or not your submission contains all the raw data required to replicate the results of  your study. Authors must share the “minimal data set” for their submission. PLOS defines the minimal data set to  consist of the data required to replicate all study findings reported in the article, as well as related metadata and  methods (https://journals.plos.org/plosone/s/data-availability#loc-minimal-data-set-definition).  For example, authors should submit the following data:  - The values behind the means, standard deviations, and other measures reported;  - The values used to build graphs;  - The points extracted from images for analysis.  Authors do not need to submit their entire data set if only a portion of the data was used in the reported study.  If your submission does not contain this data, please either upload it as a Supporting Information file or deposit  it in a stable, public repository and provide us with the relevant URLs, DOIs, or accession numbers. For a list of  recommended repositories, please see https://journals.plos.org/plosone/s/recommended-repositories.  If there are ethical or legal restrictions on sharing a de-identified data set, please explain them in detail (e.g., data  contain potentially sensitive information, data are owned by a third-party organization, etc.) and who has imposed  them (e.g., an ethics committee). Please also provide contact information for a data access committee, ethics  committee, or other institutional body to which data requests may be sent. If data are owned by a third party, please  indicate how others may request data access. | The raw data sets have been submitted as a supplementary file together with the manuscript. | NA |
| 4 | Please amend either the title on the online submission form (via Edit Submission) or the title in the manuscript so that they are identical. | The title on the manuscript has been amended to match the title on the online submission. | 1 |
| Reviewer #1 | | | |
| 1 | The authors have a well-written manuscript with clear results.  This manuscript however could be improved in some aspects. In your abstract, I suggest you rewrite your conclusion to include your key findings, as you found other genotypes of HPV in your work. | Thank you for this comment. |  |
| 2 | In the main script under your methods describing histology, could you provide some more details on how this method  was carried out? | Following a thorough history and physical examination, a punch biopsy was taken from a cervical lesion was taken for confirmation of diagnosis. After fixation in neutral buffered formalin and processing of the biopsied tissue, formalin-fixed paraffin-embedded (FFPE) tissue blocks were made. The blocks were sectioned, and 5-micron thickness slides were obtained, stained by hematoxylin and eosin (H&E), and examined by two trained pathologists to confirm or rule out CC diagnosis. | 6 |
| 3 | Under HPV genotyping, you combined two methods. Could you briefly have described how you carried out this method to aid other investigators who want to use your method? | The two methods that were used to do HPV genotyping have been described. | 7 |
| 4 | Under discussion, you highlight the involvement of HPV 35 differing in women living with HIV and those without HPV.  Could you explain why this occurs in your population of women in this study? | A paragraph has been added to show the epidemiology of HPV 35 infection in sub-Saharan Africa. | 16-17 |
| 5 | In your conclusion in the manuscript, it appears you are discussing your findings. Please rewrite to give your key findings more clarity. | The conclusion has been re-written | 18-19 |
| Reviewer #2 | | | |
| 1 | Congratulations on this commendable and well-executed study. I applaud the authors for their valuable contribution in investigating the distribution of HPV genotypes stratified by HIV status among women with cervical cancer in Tanzania—an area of significant public health importance. The manuscript is well-structured, and the study design and methodology are appropriately applied. However, upon thorough review, I have identified several areas that could benefit from improvement. Specifically, the language in certain sections could be refined to enhance clarity, precision, and scientific rigor. Additionally, some statements lack adequate supporting evidence or require further elaboration to ensure comprehensive understanding. | We appreciate your comments! |  |
| 2 | Title: Consider: Genotype distribution of Human Papillomavirus among women with cervical cancer stratified by HIV status in Tanzania  Rationale This title captures the study's focus on HPV genotype distribution, stratification by HIV status, and the geographical context, maintaining a scientific tone and precision. | Thank you.  The title has been amended as recommended. | 1 |
| 3 | ABSTRACT Methods: “confirmed CC.HIV serology” Please ensure a space is added between 'CC' and 'HIV.' Additionally, there are several instances throughout the manuscript where proper spacing is missing and should be corrected for consistency and readability. | Proper spacing has been re-checked throughout the manuscript. | 2-3 |
| 4 | Fisher's exact test was employed to compare discrete variables  The Pearson’s Chi-squared test is used more frequently than the Fisher’s exact test in the tables within the results section. Consider specifying the use of the Pearson’s Chi-squared test instead of the Fisher’s exact test where applicable. | We used Fisher’s exact test. | 8-12 |
| 5 | Introduction The introduction section is brief, with limited and repetitive citations. It lacks critical information about cervical cancer screening programs in Tanzania, such as the availability and implementation of Pap smear screening—whether these are part of national priority programs or conducted opportunistically. Additionally, consider including details on histological aspects, such as the grades of premalignant lesions (CIN1, CIN2, CIN3) and the types of malignancies (e.g., squamous cell carcinoma and adenocarcinoma) in relation to HPV infection. | Contributing factors to the high burden of CC in this region include HIV endemic populations, unavailability of organized screening programs, and the use of visual inspection with acetic acid (VIA) as a primary screening method, which has variable test performance in resource-limited settings.  In our study, we enrolled patients with invasive squamous cell carcinoma, so assessing premalignant lesions was not possible.  We have added a paragraph on the association between HPV and histology in the introduction. | 4 |
| 6 | “There are thirteen high-risk (HR) HPV genotypes (16, 18, 31, 33, 35, 39,45, 51, 52, 56, 58, 59, & 68) classified as carcinogenic.” There are 14 high-risk (HR) carcinogenic HPV genotypes, but HPV 66 has been omitted. | Subtype 66 has been added. | 4 |
| 7 | “Tanzania has an estimated 18.8 million women over the age of 15 years who are at risk of developing CC. Current estimates indicate that CC affects 10241 women per year, and 6525 of them die from it in Tanzania.” This information appears to be taken from the ICO/IARC HPV Information Centre. My concern is that the information in this organization is not updated as they do not include the latest publications on HPV and HPV related lesions, they seem to change only the year of their published report. Consider including the latest publications with the estimates. | The source of this information has been changed to the updated Globocan Cancer Observatory ( Ferlay J, Ervik M, Lam F, Laversanne M, Colombet M, Mery L, Piñeros M, Znaor A, Soerjomataram I B. Global Cancer Observatory: Cancer Today. Lyon, France: International Agency for Research on Cancer. [Internet]. IARC. 2024 [cited 2025 Feb 17]. Available from: https://gco.iarc.fr/media/covid/factsheets/834-TZA-tanzania.pdf) | 4 |
| 8 | “About 3.3% of women in the general population are estimated to have HPV-16/18 infection at any given time, and 68.0% of CC are attributed to HR-HPVs 16 or 18.” I am unable to confirm the percentages presented based on the cited publication. Please provide additional details about the three licensed HPV vaccines, including the HPV genotypes they target, and specify that Tanzania has opted for the quadrivalent HPV vaccine. | The reference for this information has been edited. | 4 |
| 9 | METHODOLOGY HIV testing and CD4 count What company and instrument was used to measure CD4 cell count? | All CD4 tests were done according to the Omega Diagnostics package insert manufacturer's instructions. | 6 |
| 10 | Histology: Please provide additional details on the histological methodology before staining the slides with H&E, including the manufacturer's information for the H&E stain. Additionally, specify how many histopathologists were involved in screening the slides for quality control purposes. | Additional information on the histological methodology before staining with H& E has been included, together with the manufacturer's information for H& E stain (Abbey Color)  Two histopathologists were involved in screening the slides for quality control. | 6 |
| 11 | Statistical analysis: This section needs improvement; the STROBE guidelines will be immensely useful for this project and your future projects. | Strobe guidelines for cross-section studies have been followed in statistical analysis reporting. | 7-8 |
| 12 | The manuscript does not explicitly specify the data management tool utilized. It would be helpful to clarify whether the data was collected and managed using Microsoft Excel (specifying the version), Microsoft Access, Redcap, or any other specific data management system. | An Excel sheet was used for data entry. | 7 |
| 13 | “The study participants' descriptions were presented using percentages for discrete variables and mean (standard deviation) for continuous variables.” The mean and standard deviation were used in the study’s results section. “Median and interquartile ranges were used where the continuous variables were not normally distributed.”  Kindly indicate which normality test was used, either the Shapiro-Wilk, Anderson-Darling, or Kolmogorov-Smirnov. Usually, the statistical tests are written in full, such as the Mann-Whitney U test and Pearson’s Chi-squared test. Please include Fisher’s exact test in this section as well. | Shapiro-Wilk test was used to test for normality.  All the statistical tests have been rewritten in full. | 7-8 |
| 14 | “The Mann- Whitney and Chi-square tests were employed to evaluate associations.”  What associations are being evaluated? | The Mann-Whitney U test was used to compare independent groups when the dependent variable was not normally distributed continuously. Pearson’s Chi-squared test and Fisher's exact test were used to compare groups when the dependent variable was discrete. | 7-8 |
| 15 | Define both single and multiple HPV infections. | Single and multiple HPV infections have been defined in the methodology section.  Multiple HPV infections were defined as an infection with two or more HPV genotypes in an individual, and a single HPV infection was defined as an infection with one HPV genotype. | 7-8 |
| 16 | “The level of significance was set at p less than 0.05.” Consider: A variable was considered significant if the p-value was < 0.05. | This sentence has been adopted as recommended, “A variable was considered significant if the p-value was < 0.05.” | 8 |
| 17 | “All analyses were done employing SPSS version 23.” Include the software’s full details, including its city and country. | IBM Corp. Released 2015. IBM SPSS Statistics for Windows, Version 23.0. Armonk, NY: IBM Corp. was used for analysis | 8 |
| 18 | Ethics approval: Include an ethics approval section with an ethics clearance reference number. | The ORCI research ethics committee reviewed and approved this study with an approval number 06/80/VOL.1 | 5 |
| 19 | RESULTS Table 1 and Table 2 It is recommended to include a superscript (number or letter) alongside the p-values in each column. This superscript will indicate which statistical test was employed to calculate the respective p-values. Then, show the statistical tests in the table footnote. | Tables 1 and 2 include a superscript alongside the p-values to indicate which statistical test was used. | 8-12 |
| 20 | Table 2 Under the 'histology' variable, consider including the other types of malignancies. Under the variable 'histological grade,' please specify that it refers to cervical intraepithelial neoplasia (CIN). “Mean CD4 count (cell/mm3) (IQR)” The word ‘mean’ is a typo, the correct word is median. | Other types of malignancies have been added under the histology variable.  The grade refers to histological grade and not CIN.  Mean CD4 counts has been changed to median CD4 count | 10 |
| 21 | I would include a table showing the prevalence of HPV infection according to HIV status. The outcome variable will be HIV status, and the dependent variables will be Any HPV, single HPV infection, multiple HPV infection, HR-HPV infection, LR-HPV infection, and probable HR-HPV.  I recommend including a 95% confidence interval (CI).  Rationale: Including a 95% confidence interval provides a measure of precision for the estimate and conveys the range within which the true value is likely to fall. It enhances the interpretability and reliability of the results, allowing readers to assess the statistical significance and variability of the findings. | In our study, LR-HPV was not detected.  A table ( Table 3) has been added to show the distribution of HPV infection (any HPV infection and single/multiple HPV infections) according to HIV status and confidence intervals. |  |
|  |  |  | 11-12 |
| 23 | Figure 1, Figure 2, and Figure 3 On the y-axis of each figure, kindly indicate HPV prevalence (%) | HPV prevalence (%) has been added in the Y axis of Figures 1 and 2 | 13-15 |
| 24 | Figure 2 and Figure 3 Include a statistical test used to obtain the p-value on the figure legend. | Fisher's exact test was used and has been added in the legend. | 13-15 |
| 25 | limitation The authors discussed some limitations. However, other limitations, such as selection bias, were not highlighted, thus could influence the results | Thank you!  Simple random sampling was used to avoid selection bias. |  |
